# Supplementary material for: Are carbon emissions trading and green financial instruments synergistic? -Comprehensive quantitative research based on content analysis
Source: PLoS One. 2024 Mar 7;19(3):e0298601. doi: 10.1371/journal.pone.0298601 (PMC10919601; doi:10.1371/journal.pone.0298601)
Supplement: S1 Data — (ZIP) [file pone.0298601.s001.zip › Data/地级市Z_F工作报告.zip!/─┌├╔╣┼/║⌠┬╫▒┤╢√╩╨/└┤╫╘╣1⁄2╓┌║┼í╛┬φ┐╦╩2╛▌═°í┐_2013.docx]

各位代表：

现在，我代表市人民政府向大会报告工作，请予审议，并请市政协委员和列席会议的同志提出意见。

一、过去五年工作回顾

本届政府任期的五年，是我市经济快速发展、社会全面进步、人民生活显著改善的五年。在自治区党委政府和市委的领导下，市政府团结带领全市各族人民，有效应对世界金融危机和国内经济下行影响，努力克服经济社会转型和矛盾易发多发等各种困难，以“美丽发展、科学崛起、共享繁荣”统揽全局，解放思想，扎实工作，全面完成了各项目标任务。呼伦贝尔已经站在了新的历史起点上。

——综合经济实力大幅提升。2012年，全市地区生产总值预计完成1335亿元，增长1.62倍。固定资产投资预计完成912亿元，增长2.75倍。地方财政总收入完成137.27亿元，增长1.39倍。地方财政总支出达到294.44亿元，增长2.1倍。三次产业结构优化为18.3：46.3：35.4，工业主导地位初步确立。农牧业综合生产能力显著提高，粮食产量实现五连增，达到113亿斤。牧业年度牲畜存栏实现四连增，达到1830万头只。工业迅猛发展，累计投资1762亿元，实施重点项目1836个，对GDP贡献率提高11.6个百分点，达到39.3%。社会消费品零售总额预计达到399亿元，增长1.48倍。游客人数和旅游收入分别达到1430万人次和215亿元，均增长了3倍。各金融机构存款余额突破1000亿元，居全区第五位。单位GDP能耗下降3.79%，完成了节能节水减排任务。

——发展基础条件明显改善。城市建设投资960亿元，实施供水、供热、道路等项目1280个，建设房屋3442万平方米，荣获“中国人居环境范例奖”。公路投资282亿元，新增7109公里，总里程达到20786公里，路网密度达到8.22，实现了嘎查村全部通公路，绥满国道阿荣旗—博克图—牙克石段建成通车，高速公路实现了零的突破。铁路投资53.4亿元，新增185.4公里，滨洲铁路复线全线贯通，两伊铁路建成通车，阿扎铁路进展顺利。民航投资4.89亿元，呼伦贝尔海拉尔机场开通国内外航线33条，跨入“百万空港”行列，满洲里机场改扩建全面完成，根河通用机场正式通航，通用机场群建设有序推进。电网投资88亿元，呼伦贝尔至辽宁500千伏直流送电通道和500千伏海北输变电工程投入使用，城乡电网供电保障能力逐步增强。水利投资37.8亿元，建成了1个水利枢纽和3个中型灌区，水库库容达到93亿立方米，农田有效灌溉面积达到369.5万亩。工业园区投资95.3亿元，配套设施日趋完善。

——改革开放取得重大进展。全面加大改革力度，森工集团和地方林业六局剥离企业办社会职能改革扎实推进，集体林权制度改革主体任务和森工集团主辅分离基本完成。呼伦贝尔电网整体划转国家电网。财政、金融、农村牧区、文化、医药卫生和事业单位等改革不断深化。引进神华集团、中国黄金、同联制药、阜丰科技和大连万达等38家国内外大型企业集团，其中世界500强企业10家。培育壮大市场主体，整合了呼伦贝尔经济技术开发区，组建了呼伦贝尔农垦集团公司，成立了呼伦贝尔城市投资公司和水务投资公司。努力提升对俄蒙开放水平，蒙古国领事馆设立工作取得突破，海拉尔国际航空口岸落地签证业务获得批准。满洲里国家重点开发开放试验区建设全面启动，中蒙跨境经济合作区前期工作稳步推进，口岸基础设施建设得到加强，通关能力提升到5000万吨和500万人次。进出口总额累计完成130亿美元。主动融入东北老工业基地，承接发达地区产业转移，累计引进国内（市外）资金2449.5亿元，实际利用外资4.2亿美元。非公经济长足发展,占GDP比重达到50%以上。

——生态环境保护与建设持续加强。全面落实草原生态保护补助奖励政策，累计发放资金10亿元。退耕300万亩，退牧3313万亩。投入6.9亿元，治理沙地452万亩，草原沙化势头得到有效遏制。实施天然林保护、三北防护林等重点工程，造林577.4万亩，森林蓄积量增加8170万立方米，达到11.3亿立方米，森林覆盖率达到51.3%，提高2个百分点。承办了第九届中国城市森林论坛，荣获“国家森林城市”称号。淘汰落后产能，关停14台小火电机组、12个小水泥厂、9个小煤矿和1家造纸企业。开展绿色、生态、和谐矿山建设，矿山企业保护生态的主动性和自觉性逐步增强。我市已被国家发改委批准为低碳试点城市。

——人民生活水平显著提高。2012年，城镇居民人均可支配收入预计达到20060元，年均增长14.1%；农牧民人均纯收入预计达到9130元，年均增长16.7%。人均消费支出达到9733元，年均增长15.2%。城乡居民人均储蓄达到2.13万元，年均增长16.5%。私家车保有量达到16.3万辆，年均增长18%。保障性住房投资209.3亿元，开发1346万平方米，惠及26.27万户家庭，城镇人均居住面积增加10平方米。城镇新增就业14.23万人，农牧民转移就业36.09万人，就业工作受到国务院表彰。社会保障覆盖面持续扩大，累计支出保障金130亿元。城镇低保月标准累计提高196元，农村牧区低保年标准累计提高1580元。扶贫开发累计投入资金10亿元，实现30万人脱贫。累计为民办实事90件，呼伦贝尔爱心家园、海拉尔火车站和中心城区供水等一批重点公共公益工程投入使用，人民群众得到更多实惠。

——社会建设发展步伐加快。各级各类教育均衡发展，中小学校舍安全工程全部完成，教育水平整体提升。创新型呼伦贝尔建设扎实推进，荣获“全国科技进步先进市”称号。“五张文化名片”影响广泛，14个图书馆和42个博物馆免费开放，大型历史话剧《拓跋鲜卑》荣获“五个一工程奖”。覆盖城乡居民的基本医疗卫生制度初步建立，基本医疗保障、基本药物供应保障、基本医疗服务和公共卫生服务体系不断完善。食品药品安全监管深入开展。人口和计生工作稳步推进。群众体育蓬勃发展，竞技体育水平不断提升。精神文明建设持续加强，承办了全国农村精神文明建设工作经验交流会，成功举办了全市第三届那达慕。民主法制建设扎实推进，累计办理人大代表议案7件、建议765件，政协委员提案1238件。行政执法和公正司法水平不断提高。加强社会管理，推进社区整合，社区服务条件明显改善。恢复乡镇苏木建制27个，建设周转房5.8万平方米，改善了乡镇干部生活工作条件。畅通信访渠道，妥善解决群众合理诉求，累计化解积案2259件。圆满完成了十八大维稳安保任务。国防和双拥工作成效突出，连续三次荣获“全国双拥模范城”称号。安全生产形势总体良好。平安呼伦贝尔创建活动深入开展，人民群众安全感明显增强。统计、审计、供销、质监、国土、物价、工商、人防、广播电视、气象、地震、残联、民族宗教、档案史志和红十字会等工作都有新进展。全市经济发展、社会进步、民族团结、边疆安宁的大好局面进一步巩固。

各位代表！过去的五年，是我市经济社会实现重大跨越的五年，是全市各族人民得到实惠最多的五年，也是为全面建成小康社会奠定坚实基础的五年，成绩来之不易。这是自治区党委政府和市委坚强领导的结果，是市人大和市政协有效监督、大力支持的结果，是全市各族干部群众攻坚克难、团结奋斗的结果，是各民主党派、人民团体、驻呼军警部队指战员及社会各界广泛参与的结果。在此,我代表市政府，向所有关心和支持呼伦贝尔经济社会发展的同志们、朋友们，表示衷心的感谢!

五年的实践，积累了宝贵的经验。一是必须坚持美丽与发展双赢。绿色是呼伦贝尔的底色，保护好这片绿色净土是我们的使命和责任。贫穷保护不了美丽，发展才是解决所有问题的关键。我们既要金山银山，更要绿水青山。二是必须坚持扩总量与调结构并重。没有足够的经济总量，就谈不上结构调整；没有科学合理的经济结构，更不可能持续做大经济总量。扩大总量是当务之急，优化结构是长远之计。三是必须坚持加快发展与改善民生同步。让群众过上幸福生活是政府一切工作的出发点和落脚点，我们一定要牢记发展依靠人民、发展为了人民，将发展成果更多更公平地惠及各族人民。四是必须坚持经济与社会统筹。没有经济发展，社会进步就缺乏支撑；没有社会进步相适应，经济发展也难以持续。我们必须做到经济与社会统筹安排、协调发展。

回顾过去五年，也要看到发展中存在的困难和问题：经济总量小，发展不足，我市仍属于欠发达地区。经济结构不优，一产不够强，二产不够大，三产层次低，可持续发展能力不强。民营经济发展不充分，县域经济活力不足，缺少发展的内生动力。生态环境保护与建设任务艰巨，基础设施瓶颈还没有根本解决，改善发展条件任重道远。部分群众生活仍然困难，改善民生压力较大。基本公共服务相对滞后，社会建设仍然薄弱。影响我市改革发展的深层次矛盾和问题仍然存在，维护稳定面临一些新情况、新问题。干部队伍中还存在作风不实、紧迫感不强和不廉洁、不作为、不敢担当、不负责任的现象，抓落实的本领和能力不足，政府自身建设有待加强。这些困难和问题，我们要高度重视，切实加以解决。

二、今后五年主要任务

党的十八大判断，我国已经进入全面建成小康社会的决定性阶段，世情、国情、党情继续发生深刻变化，我们面临的发展机遇和挑战前所未有。综合分析今后五年面临的发展形势，我们必须清醒地认识到，我市经济总量小和发展不足这个基本市情没有变，人民群众对提高生活水平和改善公共服务的期待没有变，社会转型期和矛盾凸显期这个阶段性社会特征没有变，作为边疆少数民族地区，尽快缩小与发达地区的差距、同全国全区一道建成小康社会的任务急迫而艰巨。我们一定要有形势逼人、寝食难安的紧迫感。同时，更要看到，国务院关于支持内蒙古又好又快发展若干意见、西部大开发二期规划、振兴东北老工业基地第二个十年规划、满洲里国家重点开发开放试验区等一系列政策持续发酵，我们多年奠定的基础更加坚实，各族干部群众盼发展、促发展的愿望更加强烈。加之，我市资源丰富、区位独特、产业多元、后发优势明显。这些都将成为我市经济社会持续快速发展的强大动力和有利条件。我们一定要振奋精神，攻坚克难，殚精竭虑，苦干实干，全力推动经济社会持续健康快速发展。

今后五年政府工作的总体要求是：全面贯彻落实党的十八大和市第三次党代会精神，以科学发展观为统领，按照美丽发展、科学崛起、共享繁荣的总体要求，调整经济结构，转变发展方式，以生态文明建设为基础，以投资和项目建设为载体，以改革开放为动力，全力加快新型工业化、信息化、城镇化、农牧业现代化进程，大力发展现代服务业，继续保持高于全国、全区平均水平的增长速度，努力缩小与发达地区的差距，不断增强产业发展能力、基础设施保障能力、公共服务能力和生态环境支撑能力，高度关注和改善民生，高度重视社会稳定，切实加强社会建设和政府自身建设，全面加快小康社会建设步伐。

今后五年全市经济社会发展的预期目标是：到2017年，地区生产总值年均增长17%。地方财政总收入年均增长17%。限额以上固定资产投资年均增长20%。城乡居民人均可支配收入年均增长17%。完成自治区下达的节能节水减排任务。人口自然增长率控制在5‰以内。城镇登记失业率控制在4%以内。

完成上述目标，重点做好以下工作：

（一）持续推进新型工业化。要着力构建传统产业新型化、新兴产业规模化、支柱产业多元化的产业发展新格局，稳定实现经济结构由农牧业主导型向工业主导型转变。一要扩总量。到2017年，工业固定资产投资年均增长20%，工业总产值突破2400亿元，规模以上工业增加值突破1000亿元，工业占GDP比重接近50%。二要调结构。传统优势产业重点做好煤炭和煤基精深加工，扩大电力规模，拉长有色金属链条，发展新型建材和绿色食品，形成集群联动发展格局；非资源产业重点发展生物制药、装备制造、电子信息和新能源等产业，强化科技支撑，延伸产业链条，到2017年，非资源型产业占工业总产值比重达到60%以上。三要上台阶。工业产业结构日趋合理，单位产值能耗逐步下降，产品的质量和效益明显提高，行业竞争力和产业支撑力进一步增强。到2017年，打造煤电化和农畜产品加工2个产值超千亿元的产业，形成1个产值超千亿元的核心经济开发区和3个产值超五百亿元的工业园区，建成5个以上产值超百亿元的企业。

（二）全力推进农牧林区繁荣发展。以加快农牧业产业化发展为核心，努力推动我市由农牧业大市向农牧业强市转变。到2017年，第一产业增加值达到450亿元，粮食产量达到170亿斤以上，牧业年度牲畜存栏稳定在1800万头只，畜牧业占农牧业比重达到50%以上。

提升农牧业综合生产能力。加强水利建设，强化科技支撑，推进种养业内部结构调整。到2017年，节水灌溉面积达到700万亩，土地流转1000万亩，草牧场流转2000万亩，农牧业综合机械化水平达到85%。

推进农牧业产业化。依托丰富的农畜产品，以玉米、油料、肉、乳等产业为重点，延长产业链，提升农畜产品转化能力。扶持做大玉米深加工产业，统筹谋划大型肉类深加工龙头企业，研究整合乳品加工产业。重点支持呼伦贝尔农垦集团发展，到2017年，产值突破500亿元。加强基地建设，优化空间布局，促进优势特色农畜产品生产向重点区域集中，土地草牧场流转向种养大户、合作组织和大型龙头企业集中。到2017年，农畜产品加工转化率达到65%，农畜产品加工形成的产值占工业总产值35%以上。争创国家驰名商标，打响呼伦贝尔品牌。

统筹城乡发展。加快产业集聚、人口集中、完善公共服务，扶持发展地区特色产业，加强供水、供电、道路等基础设施建设，提升教育、医疗、信息等公共服务水平，改进生产生活方式，建设生态优美、经济发展、生活舒适的新型农牧林区。

（三）强力推进新型城镇化。城镇化具有扩大投资和拉动消费的双重功能。到2017年，全市城镇化率达到70%以上，社会结构实现由农村牧区社会向城市社会的历史性转变。

加快呼伦贝尔中心城区建设。实施“东扩西优、南联北延”发展战略，扩大中心城区规模，统筹推进“一城三区一镇”一体化发展，率先将海拉尔打造成在蒙东地区具有一定影响力和辐射带动力的中心城市。到2017年，人口规模达到40万人,建成区面积达到131平方公里。把握好力度和节奏，推进新区河西、河东和东山组团建设。打造新型城市综合体和产业集成地产。抓好海拉尔老城区绿化、亮化、美化和供热、供电、小巷改造等基础设施建设。建设伊敏河景观带、东山城市功能区、西山生态景观区和城北海拉尔河湿地等项目。实施城市出口畅通工程，建成城市外环路。打造交通便捷、功能完善、宜居宜业的中心城区。

加快区域中心城市建设。满洲里、牙克石、扎兰屯等区域中心城市要突出地域特色，科学规划，提升城市品位。加强市政基础设施建设，提高城市绿化覆盖率，改善人居环境。出台人口向城区聚集政策，按照“自愿为主、来去自由”的原则，鼓励农牧民和林区群众在土地、草牧场和林地权属不变的前提下进城安居。推动森工、农垦和地方林业通过产业集聚带动人口集聚。加快城关镇和重点镇建设。规划先行，突出特色，注重实效，在基础设施、公共服务等方面推进城乡一体化，形成以城带乡、城乡一体的新型城乡关系。

（四）大力发展现代服务业。服务业是就业的最大容纳器。到2017年，第三产业增加值年均增长12%，从业人员占全社会从业人员比重达到50%以上。一要加快发展旅游业。依托自然景观、民俗文化和区位优势，打造原生态多民俗国际性旅游目的地。加快星级酒店等旅游基础设施建设，培育精品路线，加强宣传促销，促进草原森林和四季旅游均衡发展。规范经营秩序，强化执法检查和行业管理，提高服务质量和水平。二要大力发展物流业。依托海拉尔、牙克石、扎兰屯等区域中心城市，建设重点物流地区和物流港，培育大型物流龙头企业。依托口岸城市和支线机场，发展国际物流和空港物流。抢抓工业和服务业融合发展的新趋势，打造工业物流体系，提高物流业专业化水平。三要努力提升商贸业。引进大型商贸连锁企业，建设商业综合体，推动商贸业规模化、品牌化。发展电子商务、设计研发等新兴业态，提升商贸业信息化水平。抓好粮食、蔬菜、肉乳等农畜产品的加工、仓储和流通。四要着力壮大金融业。引进银行、证券、保险等金融机构，完善金融服务体系。培育地方金融机构。拓宽企业融资渠道，扶持本土企业上市。

（五）建设美丽呼伦贝尔。牢固树立尊重自然、顺应自然、保护自然的生态文明理念，像爱护眼睛一样保护好生态环境。落实国家和自治区主体功能区规划，制定完善我市森林、草原等专业规划，优化产业空间布局，严格控制开发区域和开发强度，新上项目坚决做到不符合生态环保要求的不上，不符合产业政策的不上，不符合空间布局规划的不上，真正实现点上开发、面上保护，给自然留下更多修复空间。落实大小兴安岭生态保护与经济转型规划和草原生态保护补助奖励政策，实施退牧、天然林保护二期等重点工程。到2017年，全市可利用草原的20%实现禁牧、55%实现草畜平衡。坚持每年治沙100万亩。加大生态移民力度，建立生态无人区。加大生态环境执法力度，量化生态指标，面向社会公布，接受各界监督。加强动植物保护，严惩乱砍滥伐、毁林毁草开荒、河流污染等行为。推进低碳城市建设，培育优质生态产品，逐步将生态资源优势转化为现实经济优势。

（六）继续深化改革扩大开放。改革开放是激发内力、增强活力的关键。

推动森工集团发展壮大，将其培育成林业、木材加工、旅游、矿业多元发展的大型企业集团。重点培育农垦集团，将其打造成农工商一体化的产业化航母。全面启动地方林业改革，形成新的适应市场的体制机制。完善呼伦贝尔经济开发区运行机制。加快理顺中心城新区管理体制。健全城投公司法人治理结构。逐步整合旅游资源。支持企业进行战略重组。加快医药卫生体制改革。完善市与各旗市区财政体制。扶持小微企业和本土企业发展，重视本土企业的培育，建设民营企业服务平台，确保为非公经济平等配置生产要素，公平参与市场竞争。

积极主动与东北老工业基地在产业、基础设施和政策上对接，承接发达地区产业转移。支持满洲里国家重点开发开放试验区建设，强化区域合作，放大政策效应。加快中俄蒙合作先导区政策和规划的争取，提升对外开放水平。加大招商引资力度，引进资源节约型、科技创新型、产业带动型和生态环保型项目。外贸进出口总额实现新的突破。

（七）全面加快县域经济发展。县域经济是国民经济的基础。要加强分类指导，统筹推进，促进县域经济大发展。一要认清特色资源。县域经济的本质就是特色经济。发展县域经济，要充分发挥区域特色和比较优势，认清特色资源，选准区域特色，努力走特色鲜明的产业化发展路子。二要培育主导产业。突出差异化发展和可持续发展的原则，根据优势资源来确定主导产业，集中精力将主导产业培育成为立旗立市的大产业，围绕主导产业搞配套、搞延伸、搞协作，形成产业集群。三要抓住关键环节。各旗市区要选准发展县域经济的突破口，有选择地推进城镇化、农牧业产业化、新型工业化、商贸服务业和基础设施建设，形成各自发展模式。四要突出招商引资。项目和投资是发展县域经济的重中之重，要积极争跑，承接发达地区的产业转移，带动县域经济发展。五要优化发展环境。认真落实上级政策，支持民间资本进入各个领域，使民营经济成为县域经济发展的主体力量。

（八）加强基础设施建设。通过五年的努力，从根本上解决基础设施滞后问题。公路重点推进新林北—扎兰屯—那吉屯高速公路、拉布达林—海拉尔一级公路等高等级公路建设，建设里程1036公里，与自治区基本同步实现县县通高等级公路。铁路重点推进滨洲铁路电气化改造、满洲里—伊尔施等项目，新增里程930公里，运输能力达到2.3亿吨。民航重点推进扎兰屯支线机场和一批通用机场建设，启动呼伦贝尔海拉尔机场迁建前期工作，打造市域一小时航空圈。电网重点加快500千伏和220千伏主网架建设及城乡电网改造，推进电力二期外送项目。水利重点推进诺敏、晓奇等重点水利枢纽工程和尼尔基下游30万亩灌区建设。全市节水灌溉面积达到700万亩，其中膜下滴灌达到200万亩。信息要提高通信、广电、计算机骨干网络的覆盖度，建立和完善云计算等各类信息服务应用平台。

（九）持续保障和改善民生。重点实施“五大工程”。一是收入倍增工程。多渠道增加居民收入，加大扶贫开发力度，五年脱贫15万人。实现居民收入增长和经济发展同步，到2017年，居民收入实现翻番增长。二是就业促进工程。坚持就业优先，鼓励多渠道多形式就业，促进创业带动就业，争创国家级创业型城市。到2017年，城镇新增就业17万人以上，农牧民转移就业37万人以上。三是社会保障工程。坚持全覆盖、保基本、多层次、可持续的方针，以社会保险、社会救助、社会福利为基础，以基本养老、基本医疗、最低生活保障制度和社会救助体系为重点，加快建设与经济发展水平相适应的社会保障体系。四是百姓安居工程。建立市场配置和政府保障相结合的住房制度，加强保障性住房建设与管理，从根本上改善城乡居民的居住环境。五是“菜篮子”工程。加强蔬菜基地建设，提高本地蔬菜产量；建立外来蔬菜绿色通道，减少流通环节；加大冬季仓储物流基地和平价超市建设，平抑菜价。

（十）不断加强社会建设。加强社会建设，是确保社会进步、社会和谐的重要保证。

推动科技和经济紧密结合，实施知识产权战略，加强知识产权保护。构建以企业为主体、市场为导向、产学研相结合的技术创新体系。加快教育发展步伐，优先发展民族教育，完善教育基础设施，促进均衡发展，普及规范15年国民教育，抓好校外教育，支持呼伦贝尔职业技术学院通过教育部评估验收，提升高等学校办学实力，办人民满意的教育。推动文化产业和文化事业繁荣发展，加强文化遗产保护，继续实施文化信息资源共享、草原书屋建设等文化惠民工程，基本形成覆盖城乡的公共文化服务体系。完善“蒙中西医”并重的医疗卫生服务体系，注重地方病和传染病防治，提供安全有效的公共卫生和基本医疗服务。健全食品药品监管体制和机制，实现监督网全覆盖。实施人才强市战略，推进各类人才队伍建设。发展群众体育和竞技体育，实现旗市区综合性体育场馆全覆盖。加强精神文明创建力度，提升居民文明素质。继续推进双拥模范城创建，巩固良好的军政军民关系。

加快社区建设，完善城乡社区服务功能。加强安全生产，遏制重特大安全事故。广泛开展平安创建活动，促进社会和谐稳定。建立健全政府主导、各单位及社会各界广泛参与的社会矛盾排查化解长效机制，预防和妥善处置群体性、突发性公共事件。统筹推进计生、统计、人防、红十字会、广播电视、工商、质监、气象、地震、老龄、民族宗教、残联、档案史志等工作。

（十一）切实加强政府自身建设。政府自身建设是做好各项工作的基础。一要建设廉洁政府，提高政府的公信力。严格落实党风廉政建设各项制度，强化惩治和预防腐败体系建设。增强政府工作透明度，加强对权力运行的约束，严格规定重大事项决策、项目审批、资源配置等各项程序，完善政府采购的方案细则，从源头上预防腐败滋生。强化监察审计职能，坚决查办大案要案，努力做到干部清正、政府清廉、政治清明。二要建设效率政府，提高群众的满意度。全面强化政府行政审批中心职责，减少审批事项，减化办事程序，公开办事流程，凡是属于市本级部门职责范围内的事项都要在行政审批中心办结。纪检监察、审计部门要进驻行政审批中心，实行全程监督，对效率低下、久拖不决的人和事要严肃查处。三要建设法治政府，做到依法治市。始终把依法行政贯穿于政府工作全过程，严格按照法定权限和程序行使权力。自觉接受人大依法监督和政协民主监督，高度重视舆论监督和社会监督。切实解决不作为和乱作为的问题。四要建设责任政府，提高政府执行力。时刻牢记政府就是服务，权利就是责任，始终保持勤勉敬业的状态、真抓实干的作风，切实履行好政府的职责，做到勇于担当、敢于负责，让人民满意。

三、2013年主要工作

今年是全面贯彻落实党的十八大精神的第一年，也是新一届政府的起步之年。统筹考虑当前面临的形势和任务，根据市委三届三次全委会议精神，今年全市经济社会发展的主要预期目标确定为：地区生产总值增长18%。地方财政总收入增长18%。限额以上固定资产投资增长20%。城乡居民人均可支配收入增长17%。完成自治区下达的节能节水减排任务。人口自然增长率控制在5‰以内。城镇登记失业率控制在4%以内。

完成全年目标任务，必须在保增长、调结构、强基础、惠民生和抓落实上下功夫，重点做好七项工作。

（一）以项目和投资为重点，确保工业经济持续较快发展。实施市本级重点项目100个，工业固定资产投资和规模以上工业增加值增速均达到20%。

继续做大传统优势产业。煤炭行业重点推进大唐谢尔塔拉露天矿和新左旗白音查干煤田等项目建设，确保扎尼河露天矿投产达效，全年煤炭产量超过1亿吨。电力行业重点推进华电莫旗2×35万千瓦、华能牙克石2×20万千瓦和东海拉尔2×35万千瓦热电联产等项目，启动企业自备动力车间建设，电力装机达到840万千瓦。化工行业加快神华褐煤综合利用多联产升级示范项目前期工作，开工建设中煤集团200万吨化肥等项目，争取神华陈旗1000万吨褐煤提质项目一期100万吨达产达效，形成10万吨二甲醚和110万吨尿素生产能力。有色金属行业确保驰宏20万吨铅锌冶炼投产，开工建设中国黄金多金属矿分步回收综合利用等项目，形成日处理10万吨矿石采选能力。加快蒙西、北疆等水泥项目建设，熟料产量达到720万吨。推进石油等产业发展。

着力推进非资源型产业。农畜产品加工业重点开工建设同联集团扎兰屯100万吨玉米加工等项目，确保阜丰集团200万吨玉米加工项目达产达效，玉米加工能力达到300万吨。装备制造业重点加快山东能源枣庄集团机械装备制造基地建设。生物制药业确保北方药业二期和鸿洋药业一期竣工投产，开工建设鸿洋药业二期和北方药业三期工程。推进风能、光伏和生物质发电等新能源项目。启动云计算项目。

全力加快工业园区建设。突出抓好园区项目招商。做好呼伦贝尔经济技术开发区总体规划和产业规划修编，完善开发区管理体制，抓好土地运营和融资贷款，加强园区基础设施建设，提升园区服务能力，争取晋升为国家级开发区。完善各园区基础设施条件，推进土地收储，加快伊敏煤电基地和扎兰屯岭东、阿荣旗建材、满西产业基地等工业园区建设，增强园区自我发展能力。

大力扶持小微企业发展。落实上级各项政策，搭建服务平台，完善融资担保，培育本土企业，推动小微企业健康发展。

（二）以农牧业产业化为重点，加快农牧林区发展步伐。以农牧业产业化为引领，加大投入，调整结构，提升农畜产品加工转化能力，增加农牧民收入，促进农牧林区经济发展。

培育产业化龙头企业。抓好玉米、油料、肉、乳和林果等十大产业，重点在精深加工、产业延伸和产品升级上下功夫。扶持呼伦贝尔农垦集团、同联药业、阜丰科技、雀巢和中荣等龙头企业发展，以组建呼伦贝尔肉业集团为核心，在大型龙头企业培育上取得突破。引入战略合作伙伴，通过战略重组，培育加工、流通等大型龙头企业。

推进农牧业基地建设。支持龙头企业投资建设基地，新建中荣万头肉牛育肥场等规模化标准养殖场100个，加快雀巢奶业基地建设，推进农区设施养殖基地建设，增加玉米、大豆、马铃薯等标准化种植基地，启动建设良种繁育基地。规范发展农牧民专业合作社，形成合理、稳定的利益联结机制。

加快农牧林业内部结构调整。大力发展农区畜牧业，提高农区畜牧业占全市畜牧业比重，提高畜牧业占农牧业的比重。种植业内部结构调整要以增加玉米种植面积为主，确保今年达到900万亩。加大林业内部结构调整，大力发展替代产业，改变产业单一局面。

提高农牧业综合生产能力。抓好农业综合开发，流转土地550万亩、草牧场1100万亩。加大农田水利建设投入，新增节水灌溉43万亩，其中玉米膜下滴灌达到30万亩。做好“呼伦贝尔羊肉”等3个地理标志集体商标注册。

加快新农牧林区建设。推进农牧林区水利、电网、交通、通讯、文化、教育、卫生等公益设施建设。解决15万人安全饮水问题。做好农村危旧房改造和环境整治等工作，改善农牧林区面貌。

（三）以旅游和物流为重点，提升服务业发展水平。持续做大旅游文化产业。整合旅游资源，搭建旅游投融资建设平台，市场化开发运作旅游景区。完成呼伦贝尔民族文化园、蒙兀室韦•蒙古之源民族文化园、大兴安岭森林火车游等项目建设，提升景区建设水平。打造海拉尔—满洲里—阿尔山—柴河黄金旅游线路，推进森林深度游，开发冬季冰雪旅游，启动旅游服务驿站建设，促进草原森林旅游均衡发展。加快星级酒店和旅游网络平台建设，提高旅游接待能力。建立行业管理平台，强化行业培训和监管，加大执法检查力度，提升旅游服务水平。旅游接待人数和收入均增长20%以上。建设文化创意产业园，打造新的文化旅游品牌。加快物流业发展。整合现有物流企业，启动大型物流交易市场。确保牙克石大兴安国际物流园建材和商贸区投入使用。加快建设海拉尔中俄蒙国际物流园、扎兰屯岭东商贸物流园和阿荣旗中心物流园。建设海拉尔红星美凯龙城市商业综合体、满洲里国际木材交易市场。促进金融业发展。推动金融企业增设分支机构，引进新的金融机构，加快发展小额贷款公司。提升商贸业水平。推进万村千乡市场工程和新网工程，建设2个配送中心和100个村级连锁农家店。发展研发设计、商务会展、网店快递、家政服务等新兴服务业。

（四）以生态保护与建设为重点，提升生态环境承载能力。统筹抓好天然林保护二期、重点公益林保护和退牧还草二期工程，继续落实草原生态保护补助奖励等各项政策。退牧还草415万亩、禁牧2850万亩、草畜平衡7500万亩，森林采伐量控制在110万立方米以下。完成100万亩沙区治理任务。继续开展绿色、生态、和谐矿山建设。推进东北黑土区水土流失治理和小流域治理。加强生态治理后期管护，巩固生态治理成果。加强野生动植物保护，严惩毁林毁草开荒等破坏生态环境行为。提升土地优化利用率，拓展建设用地6000公顷，完成10万亩土地项目整治。完善森林城市建设，重点实施中心城区北出口绿化。开工建设呼伦贝尔自然博物馆。

（五）以城乡基础设施建设为重点，打造新的经济增长点。

城市建设。投资339亿元，建设房屋1350万平方米，实施给排水、供热、城镇道路等251个城市基础设施项目。完善中心城新区河东组团城市设计，编制控制性详细规划，加快中心城新区河东部分建设，开工建设伊敏河景观带，建成成吉思汗大街等14条道路，确保中心文化区“三馆一湖”投入使用。建成中心城东出口和西出口道路。加快城市环路建设，建成中心城区西环路。理顺呼伦贝尔中心城新区管理体制。加大东山组团、满洲里、扎兰屯、牙克石、阿荣旗等新区建设力度。

基础设施建设。公路投资55亿元，重点推进国道那吉屯—尼尔基、省道金边壕—大兴等13条公路建设。铁路投资60亿元，重点实施阿荣旗—莫旗铁路、海拉尔—拉布达林—黑山头铁路、满洲里—伊尔施铁路和滨洲铁路电气化改造等10个项目。民航投资2亿元，开工建设扎兰屯支线机场和新右旗、莫旗通用机场，组建呼伦贝尔通用航空公司。积极开辟境内外旅游航线。水利投资15亿元以上，确保扬旗山水库蓄水，开工建设扎敦等水利枢纽和尼尔基水利枢纽下游30万亩灌区，推进晓奇、诺敏水利枢纽前期工作。电网投资18亿元，开工扎兰屯500千伏变电站工程，推进33项110千伏及以上输变电工程。加快平安城市、智能交通系统和数字城市等信息基础设施建设。

（六）以提升经济发展活力为重点，深化改革扩大开放。

支持森工、农垦等国有企业改革，深化集体林权制度改革，稳妥推进地方林业剥离企业办社会职能改革。规范国有资本经营收益征缴和国有资产运营行为。做好事业单位改革机构分类工作。深化医药卫生体制改革，加快新农合支付方式改革，完善新农合市级统筹管理，推进县级公立医院试点改革。完善市与各旗市区财政体制，深化部门预算、国库集中支付和非税收入收缴制度等各项财政管理改革。

加强与东北老工业基地在产业、项目和基础设施上的对接，提高招商引资的针对性，承接发达地区产业转移。争取将我市设立为国家开发开放与保护生态环境综合配套改革试验区。支持满洲里国家重点开发开放试验区建设，高质量做好试验区建设总体规划和专业规划。启动呼伦贝尔中俄蒙合作先导区规划和满洲里综合保税区建设。加大口岸、对外通道等基础设施建设力度，加强中俄蒙经贸文化合作。推进诺永达拉果铅锌矿和别列佐夫铁矿等境外项目建设。外贸进出口总额和口岸过货量保持稳定增长。

优化发展环境，加大金融扶持力度，鼓励和引导非公企业围绕优势特色产业搞延伸，围绕重点项目搞协作，围绕大型基地搞配套，非公经济占GDP比重保持在50％以上。

（七）以保障和改善民生为重点，提高社会建设水平。

着力保障和改善民生。在全面落实国家各项惠民政策的同时，更要全方位关注人民群众日常生活，解决好供热、供水、供电、供气等生活保障问题。妥善安排好老百姓的衣食住行、柴米油盐等日常生活，统筹抓好粮食销售、牲畜出栏、蔬菜供应和物价稳定等事关群众收入的问题，确保广大人民群众安居乐业。老城区要继续推进亮化、绿化、美化和小巷治理工作，改善人居环境。新区要重视做好居民出行、购物、上学、就医等配套工作，加快完善功能。

加快社会事业发展。推进科技创业服务中心建设，指导呼伦贝尔经济技术开发区申报国家级高新技术产业开发区，推动海拉尔科技示范园区申报国家级农业科技示范园区。促进各级各类教育均衡发展，提高教育教学质量，加快幼儿园和风雨场馆建设,做好校车的安全运营与管理。弘扬优秀的历史文化和民族文化，启动蒙古族源与元朝帝陵综合研究、内蒙古蒙古族源博物馆和呼伦贝尔民族博物院改造等项目。推进医改示范区建设，继续实施卫生信息化工程,完成呼伦贝尔市卫生信息平台及应用系统建设,建成覆盖全市的120医疗急救指挥系统。扶持蒙中医药事业发展。加强食品药品安全风险监测管理体系和应急处置能力建设。广泛开展全民健身运动，提高体育竞技水平和群众健康水平。开展应急救护培训，提高自救互救技能。发展公益慈善事业，做好残疾人和老龄工作。落实民族宗教政策。统筹抓好人口与计生、统计、审计、人才、气象、广播电视、人防、质监、地震和档案史志等工作。

促进社会和谐稳定。加强社区建设，实施农村牧区社区建设试点。推进矛盾纠纷排查化解制度化、常态化，完善信访工作机制，妥善解决群众合理诉求，把矛盾化解在源头，确保社会稳定。落实安全生产主体责任，加强森林草原防火工作，确保人民群众生命财产安全。强化社会治安综合治理，防范和打击各类违法犯罪活动，加强公共安全宣传教育和应急演练。推进国防动员和军警民边防体系建设，开展双拥模范城创建活动，支持工会、共青团、妇联等群团组织按照各自职能开展工作。

推进民主法制建设。认真执行人大及其常委会各项决议、决定，自觉接受监督。积极支持政协履行各项职能。认真办理人大代表议案、建议和政协委员提案，主动听取各民主党派、工商联、无党派人士及各人民团体的意见和建议。努力建设人民满意的政府。

各位代表！空谈误国，实干兴邦。让我们在自治区党委政府和市委的坚强领导下，紧紧依靠全市各族人民，解放思想、真抓实干，为全面建成小康社会而团结奋斗！
